# The psychology of leading together: free will beliefs, self-regulation, and self-efficacy in shared leadership

**DOI:** 10.3389/fpsyg.2026.1884373

**Published:** 2026-09-17

**Authors:** Hakki Anil Yalvaç, Elif Baykal, Fatma Daşyol, Ercan Çakir

**Affiliations:** 1 Faculty of Business and Management Sciences, Istanbul Medipol University, Istanbul, Türkiye; 2 Azerbaijan State University of Economics, Baku, Azerbaijan; 3 Suleyman Demirel Universitesi, Isparta, Türkiye

**Keywords:** free will, mediation analysis, perceived self-regulation, PLS-SEM, self-efficacy, shared leadership

## Abstract

**Introduction:**

In this study, a quantitative research model has been adopted and the possible relationships among shared leadership, perceived self-regulation, free choice, and self-efficacy have been examined.

**Methods:**

Snowball sampling surveyed 283 public and private white-collar workers online. Partial Least Squares Structural Equation Modeling (PLS-SEM) tested the model in question. Our measurement approach has strong internal consistency, convergent validity, and discriminant validity for all variables.

**Results:**

In the structural model, perceived self-regulation substantially predicts self-efficacy. While shared leadership does not have a direct significant effect on self-efficacy, it has a significant and positive effect on perceived self-regulation, and self-regulation significantly increases self-efficacy. These findings reveal that perceived self-regulation plays a full mediating role in the relationship between shared leadership and self-efficacy. In contrast, the free will variable has no significant direct or indirect effect within the model.

**Discussion:**

The results confirmed our assumption regarding the positive impact of shared leadership on employees' self-efficacy perceptions, which occurs largely through self-regulation mechanisms. In this context, the study contributes to the organizational behavior and leadership literature, while also suggesting to practitioners that they should focus on leadership approaches that support self-regulation processes to increase employees' self-efficacy.

## Introduction

Self-efficacy is one of the four psychological capacities, mentioned in positive psychology literature, leading to an increase an individual's convictions about one's own capacity to strategize plans and carry out the necessary paths to attain these plans (Maddux, 1995). As to Social Cognitive Theory, self-efficacy is effective in prioritizing one's convictions about his/her abilities over actual skill proficiency and is a significant factor that leads to high motivation, perseverance, and behavioral self-regulation (Bandura, 2001). Self-efficacy is a dynamic and context-dependent construct that profoundly affects individuals' responses to complex and challenging situations (Cabrera-Aguilar et al., 2023). Prior research indicated that elevated self-efficacy can catalyze resilience and the pursuit of ambitious goals. Individuals experiencing high levels of self-efficacy are inclined to be more motivated at work settings, they tend to feel job satisfaction more often, and experience work engagement that encourages continuous learning (Baykal, 2025; Sökmen, 2021). On the other hand, it has the capacity to act as a mediator linking individual level competencies to work-related results like job engagement, goal attainment, and job satisfaction (Cabrera-Aguilar et al., 2023).

Recent research on self-efficacy undermined that domain-specific self-efficacy, especially that is experienced in academia and white collar professions, is an important antecedent of individual level lifelong learning and resilience in the face of adversities (Shahzad et al., 2022). Furthermore, self-efficacy is associated with autonomous learning behaviors and enhanced cognitive outcomes, such as creative thinking and problem-solving, underscoring its significance in environments marked by technological complexity and uncertainty (Orakci, 2025). In this study, we also focused on free will beliefs of individuals. In the field of psychology, free will is a subjective belief system influencing cognitive processes, inner motivation, and internally oriented action (Baumeister et al., 2009). Free will refers to individuals' perceptions regarding the extent to which human activities are autonomously selected, self-determined, and internally controlled, as opposed to being influenced by external forces. In the present study, we argue that shared leadership may simultaneously strengthen employees' beliefs in personal agency (free will beliefs) and their perceived self-regulation. Although these constructs are theoretically related, free will beliefs represent a generalized belief about autonomous agency, whereas perceived self-regulation reflects the behavioral processes through which employees manage their thoughts, emotions, and actions. Together, these mechanisms may contribute to employees' self-efficacy. Thus, we composed a field research investigating the relationships between these variables via a field research conducted on white collar employees.

Organizational structures in Turkey are often a blend of collectivistic values and relatively high power distance, providing a meaningful context for the examination of shared leadership. Higher power distance may also restrict the allocation of leadership tasks although collectivistic tendencies may enhance collaboration and mutual support among employees. The cultural features of Turkey make it an ideal context to examine the relationship between shared leadership and employees' self-regulation and self-efficacy. At the same time, the results should be interpreted in this particular cultural context.

### Belief in free will

Initial research in social and moral psychology indicated that the belief in free will correlates with enhanced self-regulation, moral accountability, and prosocial conduct (Deci and Ryan, 2000). Individuals claiming the necessity of free will mostly, consider themselves as active agents that can control and direct outcomes through their own efforts and choices (Rakos et al., 2008). On the other hand, a decrease in belief in free will—often driven by deterministic indicators—has been shown to boost fraud and violence while decreasing altruistic actions, indicating that belief in free will acts as a psychological process that maintains norms of society and self-regulation (Vohs and Schooler, 2008).

The idea of having faith in free will is a vital part of a holistic agency belief system, that covers self-efficacy, locus of control, and autonomy, thus enabling individuals to exercise control of what happens to them (Bayram and Artam, 2025). A comprehensive agency belief system explains an integrated set of convictions whereby individuals consider themselves as intentional, self-directing actors capable of affecting their own life outcomes (Rakos et al., 2008). Hence, these agency beliefs strengthen individuals' sense of personal responsibility, intrinsic motivation, ethical decision-making, and psychological wellbeing (Martin et al., 2017; Monroe et al., 2017).

The current research suggests that being exposed to deterministic accounts of human conduct or robotic decision-making could reduce perceived agency, consequently altering the sense of responsibility and duty (Rakos et al., 2008). Therefore, beliefs in free will are crucial for maintaining agency, responsibility, and adaptability in contemporary social and business settings.

Positive psychology underscores free will, as agency and autonomous choice are essential for human happiness, development, and wellbeing. In this context, the belief in one's capacity to make autonomous choices fosters personal development, resilience, and the attainment of significant life achievements (Yang and Oka, 2023). Individuals who endorse beliefs in free will are more inclined to perceive experiences as opportunities for self-directed growth rather than as predetermined occurrences, which corresponds with fundamental constructs of positive psychology, including self-determination and intrinsic motivation Ryan and Deci (2006) as cited in Yang and Oka (2023). At the same time, free will beliefs are linked with sense of purpose, authentic behavior, and meaning in life, which are essential dynamics leading to wellbeing in positive psychology approach (Crescioni et al., 2016). The idea in free will fosters a profound commitment to personal goals (Stinnett et al., 2025) and amplifies individuals' sense of agency. Belief in free will empowers individuals to take responsibility for their choices and achieve optimal functioning (Baumeister, 2024). Specifically, free will beliefs are conceptualized as a higher-order agency belief, and perceived self-regulation is a proximal behavioral mechanism through which employees translate agency into goal-directed action in organizational settings that fosters inspiration, commitment, and meaningful engagement in life.

Previous research in psychology has shown that beliefs in free will are positively correlated with perceived self-regulation, but the two are conceptually distinct. Free will beliefs refer to peoples generalized beliefs that human behavior is determined by autonomous choice, intentional agency, and personal responsibility (Baumeister et al., 2009; Rakos et al., 2008). Perceived self-regulation, in contrast, is a behavioral self-management process by which individuals monitor, control and regulate their thoughts, emotions, and behaviors to achieve desirable goals (Duckworth et al., 2021; Panadero and Alonso-Tapia, 2023). Accordingly, free will beliefs are a cognitive stance toward one's agency, whereas self-regulation is the psychological process by which such agency is enacted in everyday behavior. Thus, in the present study we consider these constructs to be complementary, not interchangeable. Specifically, free will beliefs are conceptualized as a higher-order agency belief, and perceived self-regulation is a proximal behavioral mechanism through which employees translate agency into goal-directed action in organizational settings.

Moreover, beliefs about free will have been linked to employees' perceptions of personal agency, accountability, and self-directed behaviors in organizational settings (Baumeister et al., 2009). These beliefs are relevant to collaborative leadership in that participatory leadership approaches foster employees' responsibility, independence, and participation in collective decision-making (Carson et al., 2007). Moreover, those with a sense of autonomy as an actor are more likely to engage in self-regulatory behaviors, which have been consistently linked to high levels of self-efficacy beliefs (Bandura, 1991; Schunk and DiBenedetto, 2020). In the present research, the concept of free will was used as a psychologically important theoretical construct that can be related to employees' self-regulation and self-efficacy in collaborative leadership contexts.

### Shared leadership

Shared leadership is a new way of leading that spreads power, decision-making, and responsibility among many team members instead of putting all of it in the hands of one person (van Knippenberg et al., 2025). This is different from traditional vertical or hierarchical leadership, where formal leaders are in charge of and organize what the team does. In shared leadership, team members work together to share power and organize leadership roles based on their skills, the needs of the task, and how well they work together (Choudhury and Maupin, 2025). This makes the team's leadership structure more flexible and dynamic. It is an interactive influence mechanism evolving as team members support one another in achieving common goals (Xu, 2025).

A growing body of empirical research demonstrates that shared leadership is particularly beneficial in contexts that demand innovation, information dissemination, and adaptability. Research on knowledge-based teams demonstrates that shared leadership significantly enhances team innovation by promoting open communication, facilitating member interactions, and strengthening team resilience, thus supporting creative problem-solving and adaptive performance in uncertain environments (Tang et al., 2024). Studies on innovation teams show that shared leadership boosts creativity, especially when accompanied by psychological safety, cognitive motivation, and social motivation. Team atmosphere and motivation are crucial to maximizing shared leadership benefits (Sun et al., 2023).

Shared leadership may impact team diversity and innovation. Sharing leadership in multigenerational teams can increase cognitive conflict and decrease affective conflict, enhancing innovation performance by merging multiple viewpoints (Wang and Duan, 2025). These findings suggest that dispersed leadership roles increase workplace innovation by integrating talents and viewpoints.

Teamwork and tenacity in difficult situations lead to creative solutions with shared leadership (Tang et al., 2024). Through shared leadership, communication quality and collaborative coping capacities affect performance outcomes.

Many studies suggest success, but shared leadership involves compromise. Depending on task complexity and team structure, good shared leadership strategies require more effort and communication but yield rewards (Xu, 2025). In dynamic and complex situations like innovation teams or knowledge-intensive businesses, shared leadership can replace hierarchical leadership due to collective capacities and performance responsibility.

Multiple subdimensions of shared leadership show how team members allocate and perform leadership influence and functions, capturing task-related and relational aspects of team activities. Planning and organizing, which involves cooperatively setting goals, structuring tasks, and coordinating team activities, promotes shared decision-making and ownership (Alghamdi, 2024). Another subdimension, problem-solving, emphasizes critical thinking as the team identifies problems, analyzes choices, and implements solutions (Alghamdi, 2024). Support and consideration, another essential feature, includes interpersonal and affective actions like encouragement, feedback, and emotional support that build team trust and cohesion (Alghamdi, 2024). Development and mentoring explains how team members train and guide each other to retain reciprocal leadership beyond work completion (Alghamdi, 2024). Asbas et al. (2024) define task coordination as a subdimension of shared leadership that aligns tasks and responsibilities to integrate workflow. Like support and consideration, personal support emphasizes emotive interpersonal reinforcement that enhances morale and teamwork (Asbas et al., 2024). As stated by Asbas et al. (2024), shared leadership creates the necessary baseline for combining high performance with relationship processes assisting team growth and success in better in challenging situations.

### Self-regulation

Self-regulation is a psychological asset helping individuals to notice and control one's own thoughts, emotions, and reactions (Duckworth et al., 2021). Modern psychological research defines self-regulation as a dynamic, goal-oriented process encompassing cognitive, motivational, and emotional elements, rather than a fixed attribute. According to the extant literature, self-regulation helps to maintain effort, control distractions, and modify methods in the face of changing task and role demands (Panadero and Alonso-Tapia, 2023). Self-regulation is linked with planning, self-monitoring and self-evaluation promoting success.

Related literature emphasizes that self-regulation as a crucial factor connecting motivation and outcomes, especially in intricate and technology-driven contexts. Self-regulation is a psychological skill which improves autonomy and resilience in difficult circumstances, while supporting long-term and goal-oriented perspectives, as it is mainly linked with greater job involvement (Chou et al., 2024; Van der Heijden et al., 2021). Additionally, self-regulation has been recognized as a significant predictor of wellbeing, facilitating individuals in managing stress and sustaining psychological equilibrium amid uncertainty and change (Rodríguez et al., 2022).

Individuals possessing robust self-regulation abilities exhibit greater confidence in their capacity to alter outcomes, resulting in adaptive behavior and sustained success over time (Schunk, 2023). Self-regulation is a significant psychological ability facilitating learning, goal attainment, and overall happiness. One of the biggest organizational benefits of self-regulation is improved task performance. Employees who can manage impulses, sustain attention, and persist toward long-term goals can better meet performance expectations, adapt to changing demands, and stay consistent under pressure. Self-regulation ensures ethical behavior. Self-regulation helps employees follow company norms and morality in difficult situations, reducing misconduct, absenteeism, and deviance (Kamran et al., 2023). From a leadership and organizational climate perspective, widespread self-regulatory capacity fosters trust and psychological safety, since employees can rely on one another to act responsibly and predictably (Sumanth et al., 2024). Moreover, self-regulation plays a critical role in employee wellbeing and sustainability, as it enables individuals to manage workload demands, cope with stress, and prevent emotional exhaustion, thereby lowering burnout and turnover intentions (Mihalca et al., 2024). In knowledge-intensive and high-autonomy environments, self-regulation encourages employees to structure their work and maintain motivation. Self-regulation lets employees reflect on feedback, and improve continuously and foster high quality coordination and superior performance in dynamic workplaces (Moreno et al., 2025).

### Self-efficacy

Self-efficacy is the opinion that an individual contains the cognitive, motivational, and interpersonal resources essential to accomplish a certain success (Bandura, 2013). It is not a generalized self confidence attitude. It a domain specific attitude about one's convictions regarding his/her own competencies (Nwibe and Ogbuanya, 2024). It is associated with the intricacy of tasks, domains, and circumstances. It affects goal setting, forecasting, effort management, and performance challenges. High self-efficacy leads to more ambitious goals, greater tenacity, and faster recovery from mistakes since people see difficulties as learning opportunities (Baykal, 2025). High self-efficacy helps people face challenging situations, reducing stress and anxiety.

Self-efficacy boosts organization performance, learning, innovation, and adaptability. Employees with high self-efficacy are more inclined to engage in proactive problem-solving, knowledge sharing, and continuous improvement, while also demonstrating greater openness to change and technological innovation (Bandura, 2013). Self-efficacy is built through mastery, role model learning, social persuasion with feedback and reinforcement, and physiological and emotional interpretation (Juwita et al., 2023). Self-efficacy is widely seen as a crucial psychological resource that increases individual effectiveness, resilience, and long-term performance in varied organizational situations (Guo et al., 2025).

### Hypothesis

In modern firms, shared leadership—the dispersion of leadership influence among team members rather than a single formal leader—has been linked to employee self-regulation (van Knippenberg et al., 2025). Self-regulation involves setting goals, tracking progress, controlling emotions, and adapting to work and community goals. Shared leadership promotes self-regulation in teams through autonomy, mutual accountability, and peer-based feedback (Zhu et al., 2018).

In a multi-level study by Mansoor et al. (2025), it is revealed that shared leadership in teams predicted proactive goal regulation and adaptive task behaviors through psychological empowerment. Simialrly, Ali and Jiang (2025) found that shared leadership frameworks improved team self confidence and voice behavior, enabling knowledge workers to self-monitor and engage in innovative behavior. Shared leadership tasks seem to internalize performance criteria and encourage individuals to manage their own work processes.

Shared leadership enhances self-discipline and time management (Simha, 2022). Zhou et al. (2025) discovered that shared leadership increased virtual project team peer coordination, since it decentralizes control and encourages participation, employees gain intrinsic motivation and ownership, which boosts self-regulation. Shared leadership increases goal setting, emotional management, and adaptive performance through trust, reciprocal influence, and participatory governance. Delegating responsibility to teams encourages self-regulation and sustained effectiveness. Therefore, the following hypothesis is formulated:

H1: Shared leadership has a positive effect on self-regulation of employees.

Collaborative nature of shared leadership boost employees' self-efficacy, employee decision-making, coordination, and problem-solving. Active engagement in tasks brings mastery as people take responsibility and see results. They gain confidence by completing these tasks. Recent research suggests that shared leadership boosts self-efficacy through vicarious learning and empowerment (Zhu et al., 2018). Shared leadership allows employees to witness peers leading, facilitating vicarious learning. Shared leadership also encourages regular communication and knowledge sharing, which boosts learning and confidence (D'Innocenzo et al., 2021). Psychological safety boosts confidence, and autonomous behavior (Zhang et al., 2022). In shared leadership, collaboration enhances self-efficacy, according to social cognitive theory, which also emphasizes mastery, observational learning, and social impact. When exposed to shared leadership, employees are more likely to help make decisions, work together, and solve problems (Pearce, 2004). In shared leadership, team members are accountable and see the results immediately. They feel better about themselves when they accomplish. In shared leadership, Witnessing colleagues succeed in leadership roles boosts confidence in comparable situations (Widianto, 2021). Also, the collaborative nature of shared leadership promotes regular interactions and knowledge exchange, thereby fostering confidence (D'Innocenzo et al., 2021).Shared leadership boosts self-efficacy, voice, and psychological empowerment (Edelmann et al., 2020). Finally, shared leaders protect team members and these conditions diminish failure and performance anxiety. Psychological safety impacts shared leadership confidence and proactiveness (Fransen et al., 2020).

Finally, shared leadership promotes active participation, peer learning, feedback, and collaboration, enhancing employee confidence. Therefore, the following hypothesis is formulated:

H2: Shared leadership has a positive effect on self-efficacy of employees.

Shared leadership provides employees with greater opportunity to contribute to decisions, discretionary effort and collective outcomes. Participative environments promote employees' feelings of autonomy and independence as organizational agents. Perceived self-regulation is the regulation of behavior in pursuit of goals and free will beliefs are individuals' more general perceptions of personal agency and intentionality (Wu et al., 2025), Thus, shared leadership might improve employees' perceptions about the meaning of control over their behavior and organizational output. This sense of autonomy enhances self-regulation, as individuals in shared leadership environments are prompted to establish goals, track progress, and collaboratively modify behavior instead of relying on a singular authority, thereby strengthening internal regulatory mechanisms and proactive behavioral management. Research shows that shared leadership can improve self-efficacy and psychological outcomes, including teacher self-efficacy and job satisfaction. This suggests that distributed leadership roles can boost confidence in competence and effectiveness (Katitaş et al., 2025; Özdemir et al., 2025). Shared leadership promotes psychological safety and self-efficacy in team dynamics by empowering members to take initiative and express ideas (Wang and Peng, 2022) which contributes to free will perceptions of employees. Therefore, the following hypothesis is formulated:

H3: Shared Leadership has a positive effect on free will perceptions of employees.

According to Bandura's (2013) Social Cognitive Theory, environmental conditions influence individual functioning primarily through self-regulatory mechanisms rather than direct effects on psychological outcomes. In shared leadership environments employees are involved in making decisions, working together to assign duties to others and developing a greater sense of accountability for the goals of the group. These decentralized leadership practices require individuals to self-monitor their performance, self-regulate their behaviors, self-assess their progress, and self-adjust their behaviors according to the changing needs of the team. In this way, shared leadership builds the context that naturally triggers employees' self-regulation.

Perceived self-regulation is the behavioral mechanism through which employees convert participative leadership experiences into effective actions. In shared leadership settings, employees should determine their own goals, monitor their progress, regulate their emotional responses, and adapt their behavioral strategies based on feedback, instead of relying on external supervision (Duckworth et al., 2021; Panadero and Alonso-Tapia, 2023). These self-regulatory behaviors result in repeated mastery experiences, which are one of the most potent sources of self-efficacy (Bandura, 2013, Social Cognitive Theory).

Thus, the influence of shared leadership on self-efficacy is expected to be indirect, through perceived self-regulation. Shared leadership provides the organizational context in which autonomous behavioral management is promoted and self-regulation provides employees with the ability to successfully achieve work-related goals. Repeated success in self-directed performance increases the confidence of employees in their ability to perform future tasks. Accordingly, self-regulation is the proximal psychological mechanism by which shared leadership practices translate into increased self-efficacy. Therefore, the following hypothesis is formulated:

H4: Self-regulation has a mediator effect in the relationship between shared leadership and self-efficacy.

Modern organizations expect proactive individuals to shape their procedures and results. Shared leadership gives team members equal power, decision-making, and responsibility and free will beliefs of individuals has the potential to increase possible effects of shared leadership on self-efficacy perceptions of employees. In this point, Feldman et al. (2018) found that free will increases personal growth initiative, suggesting autonomous agents are more goal-oriented and proactive increasing self-efficacy perceptions of individuals.

Moreover, increased agency and control sense of control coming from free will perceptions of employees are also effective on boosting this effect on self efficacy. In shared leadership systems, perceived control affect self-efficacy (Liu et al., 2022) and this perceived control can come from free will perceptions. Therefore, the following hypothesis is formulated:

Free will acts as a mediator in the relationship between shared leadership and self-efficacy.

## Methodology

A quantitative research method was employed in this study. A survey was administered to measure participants' attitudes and perceptions regarding the study. Accordingly, 283 white-collar workers in the public and private sectors were contacted, and a four-section survey was distributed to them via an online link using snowball sampling. The first section contains questions regarding participants' demographic characteristics and professional information. The second section includes questions from the Free Will Determination Scale to measure participants' perceptions of free will. The third section contains questions related to self-efficacy, a dimension of the psychological capital concept. The fourth section contains questions from the Shared Leadership Scale. The final section includes questions related to perceived self-regulation. To protect participants' personal information and prevent biased responses, no questions were asked regarding their first names, last names, or identification details.

Before the analyses, the dataset was screened for missing values. The online survey platform required replies to all questionnaire items before submission; hence, the final dataset contained no missing values, and no imputation processes were necessary.

As the survey was distributed using a snowball sampling procedure via online networks, it was not possible to know how many people received the survey link. Therefore, a conventional response rate could not be computed. This constraint is a characteristic of chain-referral sampling methods and has been previously reported in methodological investigations.

An *a priori* power analysis was conducted using GPower 3.1 (Faul et al., 2009) to calculate the least required sample size. A minimum of 68 individuals was required based on a medium effect size (f2 = 0.15), a significance threshold of 0.05, statistical power of 0.80 and two predictors. The present study had 283 responses which was more than sufficient to identify the proposed impacts.

### Measurement instruments

**Free will determination**. The Free Will Determination scale, developed by Stroessner and Green (1990), was used to measure employees' perceptions of free will. Responses were measured using a 5-point Likert-type scale (1 = Strongly Disagree, 2 = Disagree, 3 = Neutral, 4 = Agree, 5 = Strongly Agree). Sample items include: “I will always have free will,” “My choices are limited by God,” “I have complete free will.” The scale's Cronbach's alpha value was found to be 0.87.

**Self-efficacy**. To measure employees' self-efficacy in the study, the 3-item Self-Efficacy dimension of the Psychological Capital Scale Short Form—adapted into Turkish by (Oruç 2018) and validated for reliability and validity—was used. Responses were measured using a 5-point Likert-type scale (1 = Strongly Disagree, 2 = Disagree, 3 = Neutral, 4 = Agree, 5 = Strongly Agree). Sample items include: “When I speak about my area of expertise during meetings with managers at my workplace, I feel confident,” “I am confident in my ability to contribute to the process of defining the strategies of the company where I work,” “I feel confident when I'm sharing information with my coworkers about a topic.” The scale's Cronbach's alpha value was found to be 0.93.

**Shared leadership**. The Shared Leadership Scale was adapted into Turkish and validated by Bostanci (2012). The adaptation study demonstrated satisfactory psychometric properties in the Turkish context. Responses were measured using a 5-point Likert-type scale (1 = Strongly Disagree, 2 = Disagree, 3 = Neutral, 4 = Agree, 5 = Strongly Agree). Sample items include: “Every employee of the organization plays a role in setting goals for the organization,” “At the organization, there is one person who decides what the other employees will do,” “There are interpersonal and professional ties among the organization's employees.” The scale's Cronbach's alpha value was found to be 0.91.

**Perceived self-regulation**. To measure employees' perceptions of self-regulation in the study, the 16-item Perceived Self-regulation Scale developed by (Arslan and Gelişli 2015)—which has undergone validity and reliability testing—was used. Responses were measured using a 5-point Likert-type scale (1 = Strongly Disagree, 2 = Disagree, 3 = Neutral, 4 = Agree, 5 = Strongly Agree). Sample items include: “I can easily learn a new subject,” “When I'm learning about a topic, I can easily spot new ideas,” “I can learn from my mistakes.” The scale's Cronbach's alpha value was found to be 0.90.

### Internal consistency

The results provide good internal consistency reliability across all constructs, as Table 1 illustrates. The required threshold of 0.70 is exceeded by Cronbach's alpha values, which range from 0.915 to 0.931. All constructs show adequate reliability, as evidenced by the composite reliability values (rho_a and rho_c), which are significantly higher than the minimum allowed threshold of 0.70. These results imply that the measurement model is quite dependable and appropriate for additional structural investigation.

**Table 1:** Internal consistency.

Constructs	Cronbach's alpha	Composite reliability (rho_a)	Composite reliability (rho_c)
FW	0.923	0.870	0.936
PSR	0.924	0.937	0.935
SE	0.915	0.918	0.946
SL	0.931	0.943	0.938

The results suggest that all constructs have excellent convergent validity, as Table 2 illustrates. Every Average Variance Extracted (AVE) result, which ranges from 0.636 to 0.854, is higher than the suggested cutoff of 0.50 (Fornell and Larcker, 1981). This demonstrates that a significant amount of the variance in each construct's indicators may be explained (Hair et al., 2019a). Specifically, SE and SL show very strong convergent validity, and FW and PSR also surpass the acceptable minimum limits, indicating that the measurement model is adequate.

**Table 2:** Average variance extracted (AVE).

Constructs	Average variance extracted (AVE)
FW	0.636
PSR	0.783
SE	0.854
SL	0.827

The outside loadings in Table 3 demonstrate that every measurement item has adequate indication reliability. Every loading, which ranges from 0.701 to 0.933, surpasses the suggested threshold of 0.70, indicating that each indicator significantly contributes to its corresponding construct (Hair et al., 2019a; Hulland, 1999). Specifically, SE exhibits extremely high indication dependability, and FW, PSR, and SL also regularly display high loadings. These findings offer compelling proof of the measurement model's dependability.

**Table 3:** Outer loadings.

Constructs	FW	PSR	SE	SL
FW1	0.731			
FW10	0.796			
FW11	0.772			
FW12	0.716			
FW13	0.744			
FW14	0.724			
FW15	0.785			
FW16	0.717			
FW17	0.780			
FW2	0.776			
FW3	0.732			
FW4	0.756			
FW5	0.742			
FW6	0.758			
FW7	0.763			
FW8	0.793			
FW9	0.791			
PSR1		0.802		
PSR10		0.742		
PSR11		0.789		
PSR12		0.789		
PSR13		0.794		
PSR14		0.701		
PSR15		0.782		
PSR16		0.803		
PSR2		0.812		
PSR3		0.753		
PSR4		0.767		
PSR5		0.722		
PSR6		0.742		
PSR7		0.731		
PSR8		0.786		
PSR9		0.808		
SE1			0.918	
SE2			0.933	
SE3			0.921	
SL1				0.735
SL10				0.735
SL11				0.797
SL12				0.779
SL13				0.787
SL14				0.727
SL15				0.744
SL16				0.785
SL17				0.788
SL18				0.759
SL2				0.766
SL3				0.784
SL4				0.801
SL5				0.748
SL6				0.733
SL7				0.755
SL8				0.756
SL9				0.807

The Fornell-Larcker criterion verifies that discriminant validity is proven for every construct, as Table 4 illustrates (Fornell and Larcker, 1981). Each construct shares more variance with its own indicators than with other constructs in the model, as indicated by the square root of AVE values for each construct exceeding the corresponding inter-construct correlations (Hair et al., 2019b). These findings show that the constructs measure various theoretical ideas and are experimentally distinct.

**Table 4:** Fornell-Larcker criterion.

Constructs	FW	PSR	SE	SL
FW	0.697			
PSR	0.538	0.847		
SE	0.462	0.452	0.924	
SL	0.242	0.300	0.240	0.685

The Heterotrait-Monotrait Ratio (HTMT) results demonstrate good discriminant validity across all constructs, as Table 5 illustrates. With the strongest connection found between FW and PSR (HTMT = 0.484), all HTMT values are far below the conservative cutoff of 0.85 (Henseler et al., 2015). These results show that the notions assess conceptually separate theoretical realms and are empirically distinct.

**Table 5:** Heterotrait-Monotrait ratio (HTMT).

Constructs	FW	PSR	SE	SL
FW				
PSR	0.484			
SE	0.403	0.417		
SL	0.283	0.292	0.229	

The cross-loading results verify significant discriminant validity at the item level, as Table 6 illustrates. In accordance with the criteria suggested by Chin (1998) and Hair et al. (2019a), each indication loads highest on its intended construct, and cross-loadings between alternative constructs are significantly lower. This pattern shows that none of the measurement items significantly overlap with other constructs and are all clearly related to their respective latent variables.

**Table 6:** Cross-loadings of items.

Constructs	FW	PSR	SE	SL
FW10	0.796	−0.440	−0.359	−0.268
FW11	−0.493	0.202	0.147	0.077
FW12	−0.237	0.067	0.087	−0.050
FW13	−0.273	0.074	0.094	−0.143
FW14	−0.063	0.065	0.013	−0.126
FW15	0.040	−0.041	−0.057	−0.130
FW16	−0.473	0.187	0.199	−0.044
FW17	−0.289	0.187	0.225	−0.067
FW1	−0.581	0.181	0.151	0.010
FW2	−0.698	0.256	0.232	0.130
FW3	0.732	−0.412	−0.305	−0.192
FW4	0.756	−0.418	−0.372	−0.206
FW5	0.330	−0.250	−0.191	−0.135
FW6	−0.166	0.102	0.079	−0.135
FW7	−0.368	0.171	0.134	0.066
FW8	−0.228	0.192	0.113	−0.026
FW9	0.791	−0.495	−0.447	−0.238
PSR1	−0.316	0.566	0.386	0.171
PSR10	−0.334	0.742	0.514	0.235
PSR11	−0.442	0.789	0.562	0.274
PSR12	−0.416	0.789	0.616	0.275
PSR13	−0.365	0.658	0.445	0.298
PSR14	−0.375	0.701	0.473	0.268
PSR15	−0.445	0.782	0.536	0.244
PSR16	−0.470	0.803	0.611	0.273
PSR2	−0.457	0.696	0.503	0.206
PSR3	−0.407	0.753	0.581	0.207
PSR4	−0.260	0.476	0.379	0.088
PSR5	−0.362	0.722	0.527	0.099
PSR6	−0.069	0.279	0.187	0.042
PSR7	−0.439	0.731	0.556	0.200
PSR8	−0.239	0.590	0.349	0.103
PSR9	−0.382	0.808	0.592	0.196
SE1	−0.357	0.636	0.918	0.198
SE2	−0.436	0.650	0.933	0.258
SE3	−0.481	0.713	0.921	0.210
SL1	−0.214	0.218	0.171	0.735
SL10	−0.119	0.260	0.204	0.735
SL11	−0.211	0.287	0.196	0.797
SL12	0.042	−0.027	−0.009	0.409
SL13	−0.126	0.118	0.053	0.632
SL14	−0.016	0.157	0.133	0.323
SL15	0.038	0.031	0.043	0.284
SL16	−0.203	0.225	0.191	0.785
SL17	−0.189	0.243	0.256	0.693
SL18	−0.167	0.239	0.235	0.742
SL2	−0.236	0.227	0.162	0.766
SL3	−0.205	0.212	0.186	0.784
SL4	−0.169	0.197	0.147	0.695
SL5	−0.210	0.232	0.184	0.796
SL6	−0.109	0.042	−0.016	0.627
SL7	−0.154	0.171	0.121	0.755
SL8	−0.076	0.083	0.065	0.626
SL9	−0.182	0.242	0.172	0.807

## Results

The structural model findings from bootstrapping analysis reveal a consistent but only partially supported pattern of interactions between the constructs, as Table 7 illustrates. In particular, PSR is the most important direct predictor in the model, showing a large and statistically significant positive effect on SE (β = 0.664, *p* < 0.001). On the other hand, SL does not significantly affect SE directly (β = 0.017, *p* = 0.673), indicating that its impact on SE is indirect. On the other hand, SL considerably predicts PSR (β = 0.300, *p* < 0.001), which strongly influences SE, suggesting an indirect explanatory mechanism. While FW has no significant impact on SE (β = −0.101, *p* = 0.203), the link between SL and FW is negative and only marginally significant at the 10% level (β = −0.242, *p* = 0.075). Overall, these results imply that PSR is a key mediator in the model, transferring the impact of SL to SE while FW has little effect on the structural linkages. As a result, the findings lend credence to a scenario in which SL mostly affects SE via PSR rather than a direct channel.

**Table 7:** Results of PLS-SEM.

Constructs	Original sample (O)	Sample mean (M)	Standard deviation (STDEV)	*T* statistics (|O/STDEV|)	*P* values
FW -> SE	−0.101	−0.090	0.079	1.274	0.203
PSR -> SE	0.664	0.661	0.051	12.944	0.000
SL -> FW	−0.242	−0.243	0.136	1.783	0.075
SL -> PSR	0.300	0.315	0.058	5.187	0.000
SL -> SE	0.017	0.015	0.039	0.422	0.673

### Indirect effects

The mediation study offers convincing proof of the structural model's indirect mechanisms, as Table 8 illustrates. The findings show that SL has a positive and statistically significant indirect effect on SE through PSR (β = 0.199, *t* = 4.580, *p* < 0.001), indicating that PSR is an important mediating mechanism in this connection. This result shows that SL indirectly improves SE by raising PSR, which has a significant beneficial impact on SE.

**Table 8:** Indirect effects.

Constructs	Original sample (O)	Sample mean (M)	Standard deviation (STDEV)	*T* statistics (|O/STDEV|)	*P* values
SL -> PSR -> SE	0.199	0.208	0.043	4.580	0.000
SL -> FW -> SE	0.024	0.029	0.015	1.603	0.109

On the other hand, FW does not mediate the relationship between SL and SE because the indirect effect of SL on SE through FW is positive but not statistically significant (β = 0.024, *t* = 1.603, *p* = 0.109). As a result, in the suggested structural model, FW has no role in the transmission mechanism.

The results show a full mediation effect, since perceived self-regulation has a positive and significant indirect effect on self-efficacy while shared leadership has no significant direct effect.

### Total effects

By include both direct and indirect pathways inside the structural model, the total effects results offer a thorough summary of the general correlations among the constructs. As shown in Table 9, the results validate PSR's primary and dominant role in explaining SE, showing that it has the strongest and most significant overall effect on SE (β = 0.664, *t* = 12.944, *p* < 0.001). Additionally, SL shows a significant positive total effect on SE (β = 0.240, *t* = 4.433, *p* < 0.001), which is consistent with the combined evidence of a significant indirect effect through PSR and a non-significant direct effect, indicating that its influence on SE is mostly transmitted through mediation. On the other hand, FW does not significantly contribute to SE either directly or indirectly within the model, as seen by its non-significant overall effect on SE (β = −0.101, *p* = 0.203). Further supporting the restricted involvement of FW in the structural framework is the fact that SL has a considerable impact on PSR (β = 0.300, *p* < 0.001) but neither as a predictor nor as a mediator does free will play a significant role in the model (β = −0.242, *p* = 0.075). Overall, the findings support a mediation-based interpretation of the model by confirming that PSR is the primary explanatory mechanism driving SE, while SL primarily functions through PSR.

**Table 9:** Total effects.

Constructs	Original sample (O)	Sample mean (M)	Standard deviation (STDEV)	*T* statistics (|O/STDEV|)	*P* values
FW -> SE	−0.101	−0.090	0.079	1.274	0.203
PSR -> SE	0.664	0.661	0.051	12.944	0.000
SL -> FW	−0.242	−0.243	0.136	1.783	0.075
SL -> PSR	0.300	0.315	0.058	5.187	0.000
SL -> SE	0.240	0.252	0.054	4.433	0.000

The high indirect effect through perceived self-regulation makes the overall effect of shared leadership on self-efficacy noteworthy, even while the direct effect is not.

As shown in Table 10, the standardized root mean square residual (SRMR) and the normed fit index (NFI), current criteria for PLS-SEM (Hair et al., 2019a, 2022) were used to examine model fit. The SRMR values for the saturated model and estimated model were 0.069 and 0.077, respectively, which are below the threshold of 0.08, confirming a good model fit. The NFI statistic was also 0.97 and 0.94 for the saturated and the estimated models, respectively, which are greater than the cut-off value of 0.90. Results also lend more credence to the overall fit of the proposed structural and measurement models.

**Table 10:** Model fit.

Constructs	Saturated model	Estimated model
SRMR	0.069	0.077
d_ULS	13.471	20.328
d_G	3.078	3.142
Chi-square	4250.347	4277.748
NFI	0.97	0.94

## Conclusion

This study seeks to investigate the complex connection between shared leadership (SL) and self-efficacy (SE) through the mediating roles of perceived self-regulation (PSR) and free will (FW) variables. The findings from the structural model and bootstrapping analysis indicated a consistent but selective interaction pattern among the variables.

The primary discovery of the research is the predominant and critical influence of perceived self-regulation (PSR) on self-efficacy (SE). PSR has been recognized as the most robust direct predictor in the model, with a significantly positive impact on SE (β = 0.664, *p* < 0.001). This situation suggests that individuals' capacity to regulate their cognitive and behavioral processes is an important psychological mechanism of their judgments of competence (self-efficacy).

A significant discovery that enhances the theoretical framework of the study is that the influence of shared leadership (SL) on self-efficacy (SE) is wholly indirect. The results suggest that shared leadership does not exert a direct significant influence on self-efficacy (β = 0.017, *p* = 0.673). Shared leadership significantly predicts perceived self-regulation (β = 0.300, *p* < 0.001) and establishes a notable indirect effect on self-efficacy through perceived self-regulation (β = 0.199, *p* < 0.001). The data provide a complete mediation effect; specifically, shared leadership may contribute to individuals' views of self-efficacy solely by activating or cultivating their self-regulation abilities.

Conversely, it has been noted that the free will (FW) variable used in the model exerts a minimal influence on structural relationships. The analyses have indicated that free will has no direct influence on self-efficacy and is not a mediating factor in the link between shared leadership and self-efficacy. The negative and slightly significant correlation between shared leadership and free will (β = −0.242, *p* = 0.075) indicates that this variable is not a decisive factor within the existing institutional framework.

In conclusion, this research has put forward that self-regulation is a crucial mediator in the conversion of shared leadership mechanisms into individual results, specifically self-efficacy. In a corporate setting, the efficacy of shared leadership techniques relies not only on the delegation of authority but also on the degree to which this process facilitates employees' self-regulation mechanisms. Future research may examine whether free will beliefs operate as higher-order agency beliefs that indirectly facilitate the development of self-regulatory behaviors under participative leadership conditions.

In addition, the study has certain limitations. First, the study used an internet survey with a snowball sampling method that would restrict the generalizability of the results. Participants were not recruited using probability-based sampling but via professional and personal contacts. Thus, the sample may not be fully representative of the larger population of employees in many industries, organizational structures, and cultural settings. Therefore, the results should be interpreted with caution.

Second, participation was voluntary which may have resulted in a self-selection bias. Those who were more interested in leadership or more psychologically involved may have been more willing to participate. Anonymity and confidentiality were emphasized to reduce potential bias but self-selection cannot be avoided.

Another weakness of this study is the research design of the cross-sectional study. Since all variables were measured at the same point in time, causal relationships among shared leadership, perceived self-regulation, free will beliefs, and self-efficacy cannot be established. Therefore, the observed mediation effects should be interpreted as statistical relationships consistent with the proposed theoretical framework, not as definitive causal mechanisms. Future longitudinal or experimental studies are warranted to further explore the temporal ordering and causal relationships between these constructs.

The study was also conducted just with Turkish white-collar employees. Cultural features such as relatively high power distance and collectivistic attitudes may affect employees' perceptions of shared leadership, self-regulation, and self-efficacy. Thus, the results may not be directly transferable to organizations in various cultural contexts. Future research should validate the suggested model in other countries and cultural contexts to test the robustness and cross-cultural generalizability of the results.

### Discussion

The present study examined the possible associations among shared leadership (SL), perceived self-regulation (PSR), free will (FW), and self-efficacy (SE), testing the possible the mediating mechanisms. The findings provide preliminary contributions to the literature on leadership and organizational behavior. First, studies reveal self-control best predicts confidence. Social Cognitive Theory (Bandura, 2013) posits that self-regulation has the potential to effect individual level perceptions of efficacy. Our findings are consistent with previous studies on employee self-regulation perceptions (Liu et al., 2014; Schunk, 2023; Doménech et al., 2024; Mansoor et al., 2025; Zhou et al., 2025), suggesting that individual self-regulation may acts as a self-motivating mechanism, being associated with higher levels of self-efficacy. Second, our study is consistent with the existing literature indicating the significance of shared leadership on self efficacy (Lyons and Bandura, 2019; Seymour, 2017). Our present study is also supportive of studies indicating the absence of a statistically significant direct association between shared leadership and the existence of a clear indirect effect, highlighting the significance of internal psychological processes in comprehending the impact of shared leadership on individuals as in the studies like Hans and Gupta (2018) and Katitaş et al. (2025). Third, our model also showed that free will beliefs were not significantly associated with the proposed relationships between shared leadership and self-efficacy. Notably, there were no effects involving free will beliefs, suggesting that generalized agency beliefs may be more distal than perceived self-regulation, a proximal behavioral mechanism directly involved in goal-directed action. Thus, even if shared leadership may enhance employees' sense of agency, changes in self-efficacy appear to be more closely associated with improvements in self-regulation, rather than changes in generalized beliefs about free will (Baumeister et al., 2009; Crescioni et al., 2016).

On the one hand, previous studies like Monroe et al.'s (2017) research on self-regulation had a similar explanation for revealing organizational results better than abstract beliefs like free-will perception. Our findings provide preliminary evidence for this assumption that shared leadership may contribute to self-efficacy through self-regulation. Our results are parallel with the results of the studies revealing the fact that self-regulation has been associated with ethics, productivity, and adaptability (Kamran et al., 2023; Sumanth et al., 2024). Thus, the observed mediating effect appears to be conceptually meaningful; the perceived self-regulation revealed in this study is conceptually significant and relevant to modern organizational practices.

To sum up, this study contributes to four important points. It shows that perceived self-regulation is positively associated with self-efficacy. Second, full mediation shows that shared leadership may indirectly contribute to self-efficacy through self-regulation. Third, suggest the potential importance of self-regulatory systems in translating shared leadership practices into psychological outcomes.

### Managerial implications

This study has several ramifications for managers and companies who wish to boost employee confidence and performance. The data show that shared leadership cannot directly boost employee self-efficacy. It must empower employees to control themselves to succeed. Thus, managers should encourage decentralized and participatory leadership and teach employees how to create goals, monitor their behavior, and adjust it.

Second, firms should fund self-regulation training. Scheduling, meditation, and sensitivity training may assist workers in gaining confidence about themselves by making it easier for them to deal with their jobs and feelings. Management skills are better than abstract autonomy without free will. Finally, common leadership styles ought to encourage accountability and cooperation in firms that move quickly.

## Data Availability

The original contributions presented in the study are included in the article/supplementary material, further inquiries can be directed to the corresponding authors.
